# VIRMA promotes the progression of head and neck squamous cell carcinoma by regulating *UBR5* mRNA and m6A levels

**DOI:** 10.17305/bb.2024.10358

**Published:** 2024-10-01

**Authors:** Chunyu Zhu, Yameng Cheng, Yao Yu, Yanning Zhang, Guiyun Ren

**Affiliations:** 1 Department of Oral and Maxillofacial Surgery, School of Stomatology and Stomatological Hospital, Hebei Medical University, Shijiazhuang, China; 2 The Key Laboratory of Oral Medicine in Hebei Province, School of Stomatology and Stomatological Hospital, Hebei Medical University, Shijiazhuang, China; 3 Hebei Provincial Clinical Research Center for Oral Diseases, Shijiazhuang, China

**Keywords:** Head and neck squamous cell carcinoma (HNSCC), N6-methyladenosine (m6A) methylation, vir-like m6A methyltransferase associated (VIRMA), proliferation, invasion, biomarker

## Abstract

Head and neck squamous cell carcinoma (HNSCC) is a globally prevalent and lethal cancer form whose precise mechanisms remain incompletely understood. Increasing evidence suggests that N6-methyladenosine (m6A) plays a crucial role in cancer progression. This study aimed to explore the biological function of m6A modification and vir-like m6A methyltransferase associated (VIRMA) in HNSCC. We conducted an analysis of VIRMA expression in HNSCC cells using The Cancer Genome Atlas (TCGA) database and employed reverse transcription quantitative polymerase chain reaction (RT-qPCR) and western blotting to assess its expression levels in HNSCC cell lines. Additionally, m6A levels in HNSCC cells were quantified, and the correlation between VIRMA expression levels and the clinical and pathological features of other genes was analyzed. Upon knocking down VIRMA levels, we assessed HNSCC cell proliferation, migration, and invasion and validated downstream genes using RT-qPCR and western blot. Our findings suggested that VIRMA, as an m6A-related regulator, may significantly influence HNSCC progression by regulating ubiquitin protein ligase E3 component N-recognin 5 (*UBR5*) through m6A modification. Therefore, VIRMA may serve as a prognostic biomarker.

## Introduction

Head and neck squamous cell carcinoma (HNSCC) is the seventh most prevalent cancer worldwide, with over 500,000 new cases reported annually [1]. Despite progress in multifaceted treatments encompassing surgical interventions, radiotherapy, chemotherapy, and the latest strides in immunotherapy, HNSCC frequently culminates in fatality. This necessitates the development of innovative and efficacious therapeutic strategies to ameliorate survival outcomes in HNSCC [2]. m6A has been implicated in causing many human diseases, including tumorigenesis [3]. The methylation of m6A is regulated by the RNA methyltransferase complex (writers), the RNA demethylases (erasers), and the m6A readers. The phrase “m6A writers” typically refers to the m6A methylase complex, which consists of various proteins, including methyltransferase-like 3 (METTL3), methyltransferase-like 14 (METTL14), Wilm’s tumor-associated protein (WTAP), RNA-binding motif protein 15 (RBM15), as well as its paralog RBM15B, and KIAA1429, also known as vir-like m6A methyltransferase associated (VIRMA) [4]. METTL3 is the initial identified methyltransferase with a catalytic unit, and functions as the principal catalytic nucleus [5]. VIRMA is a virus-like m6A methyltransferase-associated protein, and its crucial role in the m6A methyltransferase complex was discovered by the Schwartz lab [6]. Knocking down VIRMA would result in an approximately fourfold decrease in the m6a peak, with a more significant impact than knocking down either METTL14 or METTL3 [7]. In contrast, alpha-ketoglutarate-dependent dioxygenase alkB homolog 5 (ALKBH5) and fat mass and obesity-associated protein (FTO) serve as meticulous erasers, reverting the m6A methylation [8]. Proteins binding to m6A are commonly termed “readers.” These encompass YT521-B homology (YTH)-domain family proteins, namely, YTHDF1, YTHDF2, and YTHDF3, along with YTH-domain proteins, specifically YTHDC1 and YTHDC2. These proteins recognize and bind to RNA modified with m6A, affecting various events in downstream RNA processing and metabolism [9, 10]. These readers specifically bind RNA modified with m6A and regulate mRNA splicing, export, stability, and translation [11].

There is increasing evidence that m6A methylation is likely a key regulatory factor in various post-transcriptional gene regulation processes, such as RNA splicing, stability, export, and degradation [12]. A plethora of recent scholarly investigations have delineated the pivotal function of the m6A apparatus in a diverse array of human malignancies. For instance, STM2457, a recently unearthed inhibitor of METTL3, has demonstrated significant antineoplastic efficacy against leukemia in both laboratory and animal models, thereby affirming the potential of METTL3 as a viable target in anticancer therapeutics [13]. Moreover, in an investigation concerning epithelial ovarian cancer, ALKBH5 was found capable of attenuating autophagy, thereby fostering tumor proliferation and invasion by modulating the mRNA stability of Bcl-2 [14]. The precise functions of VIRMA in HNSCC and the mechanisms underlying its regulation are, however, still poorly understood.

This study aimed to reveal the roles of VIRMA in HNSCC and its potential regulatory mechanism. VIRMA is significantly upregulated in the SUNE-2 and CAL-27 cell lines and shows a positive correlation with m6A methylation. Spearman correlation analysis revealed that VIRMA can interact with ubiquitin protein ligase E3 component N-recognin 5 (*UBR5*). Therefore, our study suggests that VIRMA can promote the proliferation, migration, and invasion of HNSCC through its involvement in m6A-mediated regulation of *UBR5*. VIRMA holds potential as a diagnostic and therapeutic biomarker.

## Materials and methods

### Bioinformatics analysis

The transcriptomic data and clinical information for analysis of HNSCC were downloaded from The Cancer Genome Atlas (TCGA) database (https://www.cancer.gov/ccg/research/genome-sequencing/tcga). Differential analysis of gene expression between tumor and normal mucosal tissues was executed through the use of the R package DESeq. The criterion for selecting differential genes was defined as a fold change greater than 2 and an adjusted *P* value less than 0.05. R studio was used to process RNA transcriptome data into FPKM values and generate box-type maps to analyze differences in the expression of m6A-related genes between tumors and adjacent normal tissues. HNSCC patients were divided into two groups according to the expression of VIRMA, namely the high expression group and the low expression group. The transcriptome data of the two groups were analyzed for differential expression, and the differentially expressed genes were enriched by the Kyoto Encyclopedia of Genes and Genomes (KEGG) and Gene Ontology (GO) using the DAVID database (https://davidbioinformatics.nih.gov). The online database UALCAN (https://ualcan.path.uab.edu/) was used to analyze the expression level of VIRMA in 24 different types of tumor tissues, as well as the cancer stage, tumor grade, and nodal metastasis status of VIRMA in HNSCC. The expression of VIRMA in tumor and normal tissues was analyzed using the Human Protein Atlas (HPA) online database (https://www.proteinatlas.org/). Spearman correlation analysis was performed between all genes in the tumor group and VIRMA using R studio, and the top ten genes with the highest correlation coefficient and *P* value < 0.05 were selected.

### Cell culture

The HNSCC cell lines used in this study included CAL-27, SUNE-2, and normal human oral keratinocytes. HOK cell lines were purchased from Procell Life Science & Technology (Wuhan, China). CAL-27 cell line, SUNE-2 cell line, and HOK cell line were cultured in a prepared medium. The culture medium was formulated from Dulbecco’s Modified Eagle’s Medium (DMEM, Gibco, USA), enriched with 10% Fetal Bovine Serum (FBS; Vivacell, Israel), and fortified with 1% penicillin and streptomycin (Meilunbio, Dalian, China). All cell lines underwent identification via short tandem repeat (STR) analysis (Procell, Wuhan, China) and were subsequently cultivated in an incubator (Thermo, USA), under conditions of 37 ^∘^C and a 5% CO_2_ concentration.

### Cell transfection

The lentivirus preparation kit required for transfection was purchased from Obio Technology. SUNE-2 cells were inoculated into 24-well plates and started when the cell density reached 50% according to the lentivirus guidelines of the manufacturer (Obio Technology, Shanghai, China) for transfection. The negative control vector sh-NC and the interference group sh-VIRMA were transfected into SUNE-2 cells. Three days after transfection, 5-µg/mL purinomycin (Meilun Bio, Dalian, China) was added for two weeks to screen for stable knockout cells.

### Reverse transcription-quantitative polymerase chain reaction (RT-qPCR) and real-time quantitative PCR

Total RNA was isolated from SUNE-2 cells employing the RNA easy reagent (Beyotime, China). Before the reverse transcription process, a gDNA wiper Mix (Vazyme, R323-01, China) was utilized to eradicate any remaining genomic DNA. Subsequently, the RNA was transcribed in reverse into cDNA, adhering strictly to the guidelines provided by the manufacturer. (Vazyme, R323-01, China). RT-qPCR was carried out on a StepOnePlus Real-Time PCR system (Thermo Fisher, USA) with ChamQ Universal SYBR qPCR Master Mix (Vazyme, Q711, China). Data analysis was conducted employing the 2^−ΔΔCT^ method, and the experimental procedures were replicated thrice for robustness.

### Western blot

Total cellular proteins were extracted via incubation with RIPA lysis buffer (Invent, USA), supplemented with a 1% protease inhibitor cocktail, under chilling conditions for 30 min. The protein concentrations were then meticulously quantified utilizing BCA protein assay kits (Solarbio, Beijing, China). Protein samples of identical quantities underwent separation via SDS-PAGE and were transferred onto PVDF membranes. (Millipore, USA). After sealing the PVDF membrane with a protein-free rapid sealing solution, the specified primary antibody was incubated at 4 ^∘^C overnight (Proteintech, Wuhan, China). After incubation with the secondary antibodies corresponding to the primary antibodies used, the blots were measured using an Odyssey Dual-Mode Infrared Imaging System (LI-COR, USA,) and visualized using an imaging system.

### Cell Counting Kit-8 (CCK8) experiment

Cell proliferation was evaluated using the Cell Counting Kit-8 (CCK8) assay. The SUNE-2 cells that were transfected were seeded into 96-well plates, with a cell density of 3 × 10^3^ cells per well. Following this, they were incubated in an environment with a 5% CO_2_ atmosphere at 37 ^∘^C for 2 h while being shielded from light. At subsequent, at 24, 48, 72, and 96-h intervals, 10 uL of CCK8 reagent (Meilunbio, Dalian, China) was introduced to each well. Thereafter, the OD values were measured at 450 nm using an enzyme labeler (BioTek, USA).

### EdU experiment

The selected stable cell line was employed to elucidate the impact of VIRMA on SUNE-2 proliferation, employing Meilunbio’s EdU-555 cell proliferation assay kit by the manufacturer’s guidelines. Thereafter, the cells were cultured in a six-well dish. (3×10^4^ cells/well), incubated with 10-uL EdU reagent for 1 h, fixed the cells in 4% paraformaldehyde for 30 min at room temperature. Following this, the cells were washed three times with PBS that contains 3% BSA for 3 min each time to remove excess paraformaldehyde. Then, 3% triton was added to each well and incubated at room temperature for 10–15 min, followed by washing with PBS containing 3% BSA. The preconfigured click reaction mixture was added to each well and incubated at room temperature, shielded from light for 30 min. Subsequently, 1X Hoechst 33342 solution was added to each well and incubated at room temperature and away from light for 10 min. The fluorescence was visualized using a fluorescence microscope, where Hoechst 33342 labeled the cell nuclei as blue and EdU labeled the proliferating cells as red.

### Transwell experiment

The transwell experiment utilized a 24-well plate featuring chambers with 8-µm apertures (Corning Corporation, USA). The SUNE-2 cells that were transfected were prepared in a cell suspension with a density of 3 × 104 cells using the DMEM medium that was serum-free and planted in the transwell upper chambers with a matrigel (Corning, USA) coating on the insert membrane. DMEM containing 20% FBS at 600 µL per well was injected into the lower layer of the transwell chamber as a chemical attractant. After incubating in the cell incubator for 24 h, the cells were fixed by adding 4% paraformaldehyde to the lower chamber for 30 min. Finally, each hole was treated with 600-uL 1% crystal violet solution for 30 min, wiped with a cotton swab, observed under a microscope, photographed, and counted.

### Methylated RNA immunoprecipitation (MeRIP)

The EpiQuik^TM^ CUT&RUN m6A RNA enrichment (MeRIP) kit (P-9018, Epigentek, USA) was used to detect m6A levels in *UBR5*. After 10-µg total RNA and 500-ng positive control oligonucleotides were mixed, RNA fragments containing m6A were captured by an anti-M6A antibody (#A-1801, EpigenTek). Non-immune IgG was used as a negative control. The enriched RNA was purified and fluorescence quantified for the comparison of enrichment times.

### Colony forming assay

The SUNE-2 cells that were transfected were suspended into single cells and subsequently inoculated into a culture plate with six wells at a density of 1000 cells in each well. The culture medium was changed every two days after the cells adhered to the wells. Following a continuous cultivation period of 14 days, the clonal culture process was concluded. The used medium was then discarded, each aperture washed with PBS, 4% polyformaldehyde added to fix the cells, and let stand for 30 min at room temperature. The fixed cell clones were stained with 0.1% crystal violet (Solarbio, China) at room temperature for 30 min. The results of clone formation were photographed and counted.

### Wound healing assays

Transfected SUNE-2 cells (4×10^5^ per well) were evenly distributed into 6-well plates, and incubated overnight to grow to confluent monolayers. Subsequently, a sterile 200 µL pipette tip was used to create a scratch. Phosphate buffer saline was used to wash away floating cells and serum-free DMEM was added. Images were taken by microscope at 0, 24, and 48 h and the scratch area was calculated by Image J software.

### RNA stability assays

To detect RNA stability in SUNE-2 cells, actinomycin D was added to transfected cells (10 µg/mL; MCE, USA). RNA was extracted at 0, 6, and 12 h after actinomycin addition and detected by RT-qPCR.

### m6A RNA methylation quantification assay

Total RNA was extracted from cells by RNA easy reagent (Beyotime, China), per the manufacturer’s guidelines. The EpiQuik m6A RNA Methylation Quantification Kit (colorimetric; P-9005, Epigentek, USA) was utilized to ascertain the m6A content in total RNAs, again adhering to the manufacturer’s protocol. The level of m6A was evaluated employing colorimetric techniques, by gauging the OD at a wavelength of 450 nm.

### Cell cycle assay

A total of 1-mL single-cell suspension was collected from each group following treatment with EVs. The cells were washed with ice-cold PBS and fixed with 70% alcohol at 4 ^∘^C for 24 h. Following washing twice with ice-cold PBS, the cells were suspended in 100-µL PBS, and 1-mL propidium iodide (BD Biosciences) was added to the suspension for staining at 4 ^∘^C for 30 min before cell cycle detection with an FC-500 type flow cytometer (Beckman Coulter, Inc.). The data were analyzed using the Multicycle AV software version 275 (Phoenix Flow Systems, Inc.). The proliferation index (PI) was calculated using the following formula: PI ═ (S + G2/M)/ (G0/1 + S + G2/M) × 100%.

### Methylation analysis

Utilizing the SRAMP tool (http://www.cuilab.cn/sramp), we scrutinized the m6A modification sites within the *UBR5* gene, showcasing those possessing the highest degree of reliability. In addition, through the smart app platform (http://www.bioinfo-zs.com/smartapp/), we examined the expression of Cytosine-phosphate-Guanine (CpG) methylation within *UBR5* across a spectrum of tumors, with a particular emphasis on HNSCC.

**Figure 1.**
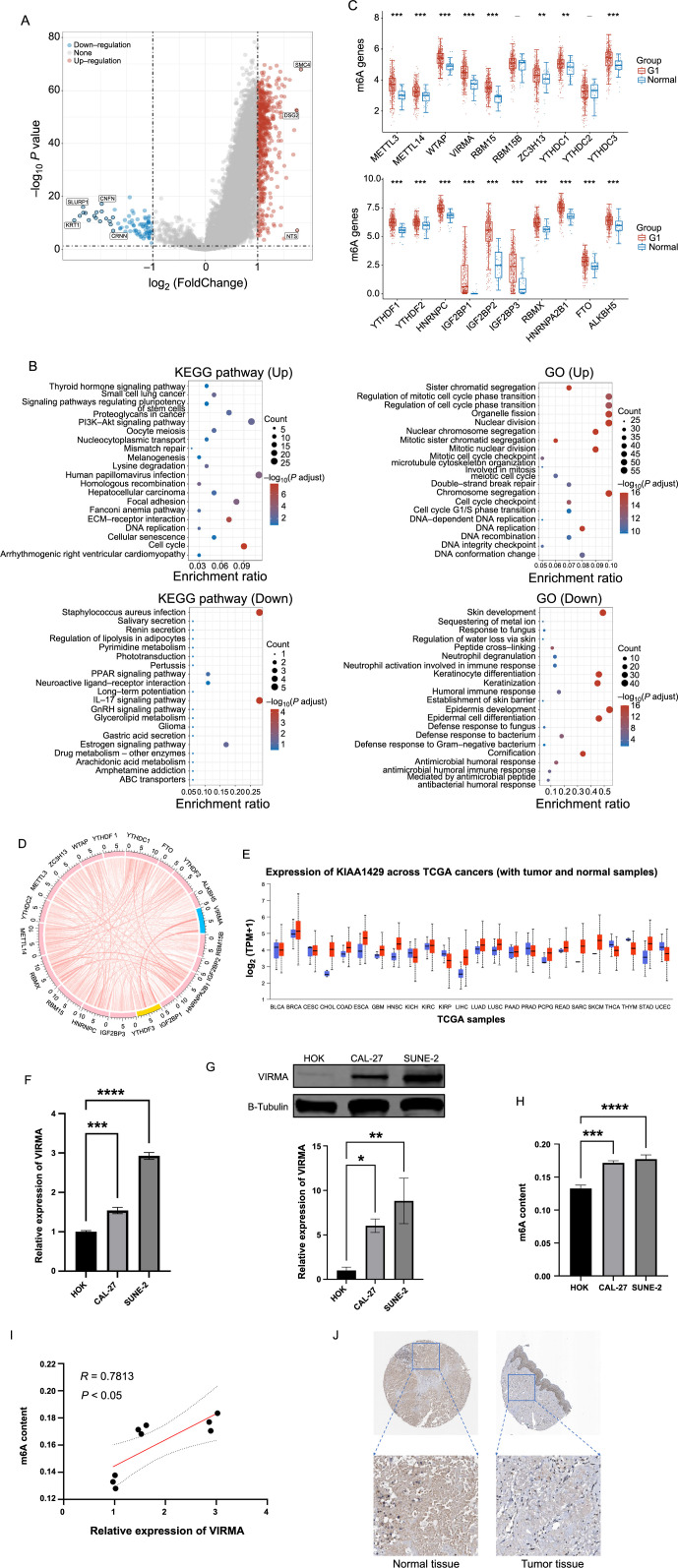
**VIRMA is highly expressed in HNSCC and associated with m6A levels.** *P < 0.05, **P <0.01, ***P < 0.001. (A and B) The differences between the standard HNSCC cohort and the tumor group in the TCGA database were analyzed, the differentially expressed genes were enriched and the KEGG pathway was involved; (C) The expression levels of 20 genes related to m6A were assessed in the TCGA database; (D) Spearman correlation analysis was conducted on 20 genes related to m6A; (E) Using the UALCAN online database (https://ualcan.path.uab.edu/), VIRMA was evaluated in 24 common tumor tissue expression types; (F) The VIRMA expression level in HOK, CAL-27, and SUNE-2 cells was measured using RT-qPCR; (G) The western blot was used to determine the protein expression levels of VIRMA in HOK, CAL-27, and SUNE-2 cells; (H and I) HOK, CAL-27, and SUNE-2 cells were subjected to m6A RNA methylation analysis to quantify the methylation level and examine its correlation with VIRMA expression. The findings suggested a positive association between the two variables; (J) Using the online database of the HPA, VIRMA expression was higher in tumor tissues than in normal tissues. HNSCC: Head and neck squamous cell carcinoma; VIRMA: Vir-like m6A methyltransferase associated; RT-qPCR: Reverse transcription-quantitative polymerase chain reaction; HPA: Human Protein Atlas; TCGA: The Cancer Genome Atlas; KEGG: Kyoto Encyclopedia of Genes and Genomes.

**Figure 2.**
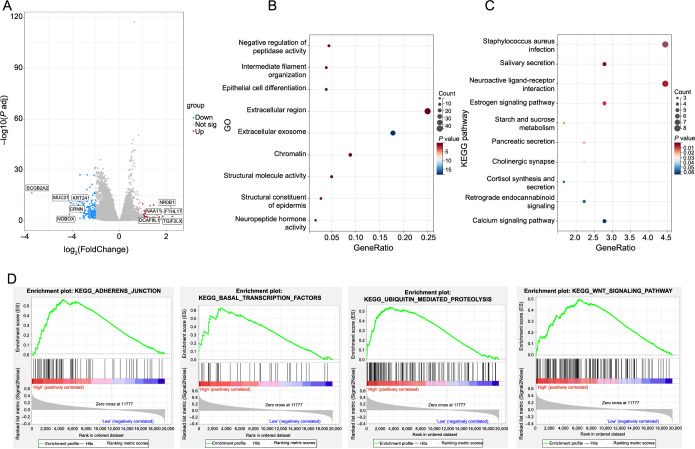
**GO, KEEG, and GSEA analyzed the biological function and signaling pathway of VIRMA.** (A) The median VIRMA expression was used to analyze the difference; (B and C) GO and KEEG analysis results of HNSCC; (D) GSEA enrichment results of HNSCC. HNSCC: Head and neck squamous cell carcinoma; VIRMA: Vir-like m6A methyltransferase associated; GO: Gene Ontology; KEGG: Kyoto Encyclopedia of Genes and Genomes; GSEA: Gene Set Enrichment Analysis.

### Ethical statement

The data sourced from the public database are freely accessible, therefore, this study was not required to obtain authorization from a clinical ethics committee. The study adhered to the relevant regulations of the public database.

### Statistical analysis

All the data were displayed as mean ± SD. Experiments were independently repeated three times. The statistical analysis was conducted with GraphPad Prism version 9.5 (GraphPad, San Diego, CA, USA). A two-tailed Student’s *t*-test was employed for group comparisons, whereupon a *P* value of less than 0.05 was considered statistically significant.

## Results

### VIRMA is highly expressed in HNSCC and associated with m6A levels

HNSCC RNA-seq data was downloaded from the TCGA database and differences in RNA-seq between the tumor group and the normal group were analyzed (Figure 1A). The differentially expressed genes were further subjected to GO and KEGG enrichment analysis. We found that most of these genes were associated with cell cycle, DNA replication, nuclear division, and mitosis (Figure 1B). Subsequently, we extracted the m6A-related genes from the differentially expressed genes and identified 20 genes that were highly expressed in HNSCC (Figure 1C). Among these genes, VIRMA had the strongest correlation with YTHDF3, with a correlation coefficient of 0.777 (Figure 1D). Previous studies have shown that m6A plays a critical role in the development of various cancers [3]. Knocking out VIRMA results in a four-fold reduction in the peak value of m6A, which is more significant than knocking out only METTL3 or METTL14 [7]. Furthermore, using the UALCAN online database, we found that VIRMA was predominantly highly expressed in 24 types of tumor tissues (Figure 1E). To validate the expression of VIRMA in HNSCC, we conducted RT-qPCR and western blot analyses on mRNA and protein, correspondingly, and found that VIRMA was highly expressed in SUNE-2 and CAL-27 cell lines compared to HOK cells (Figure 1F and 1G). Additionally, we observed that the m6A levels were higher in tumor cells compared to normal cells and showed a positive correlation (Figure 1H and 1I). To observe the expression of VIRMA in tissues, we downloaded immunohistochemical images of normal oral mucosal tissues and oral squamous cell carcinoma from the online database of the HPA. The results showed that VIRMA was positive in tumor tissue compared to normal tissue (Figure 1J).

### GO, KEEG, and GSEA analyzed the biological function and signaling pathway of VIRMA

To investigate the biological function of VIRMA, we conducted GO and KEGG enrichment (Figure 2A) through differential analysis of median VIRMA expression. The GO analysis revealed that VIRMA’s main functions encompassed epithelial cell differentiation and structural molecule activity (Figure 2B), while the KEGG analysis indicated that VIRMA played a role in neuroactive ligand–receptor interaction (Figure 2C). Moreover, the GSEA analysis indicated that VIRMA participated in the WNT signaling pathway and mediated ubiquitin-mediated proteolysis (Figure 2D). These results suggest that VIRMA may be involved in the proliferation and metastasis of HNSCC.

### Knocking down VIRMA may decrease the proliferation, invasion, and migration of HNSCC

**Figure 3.**
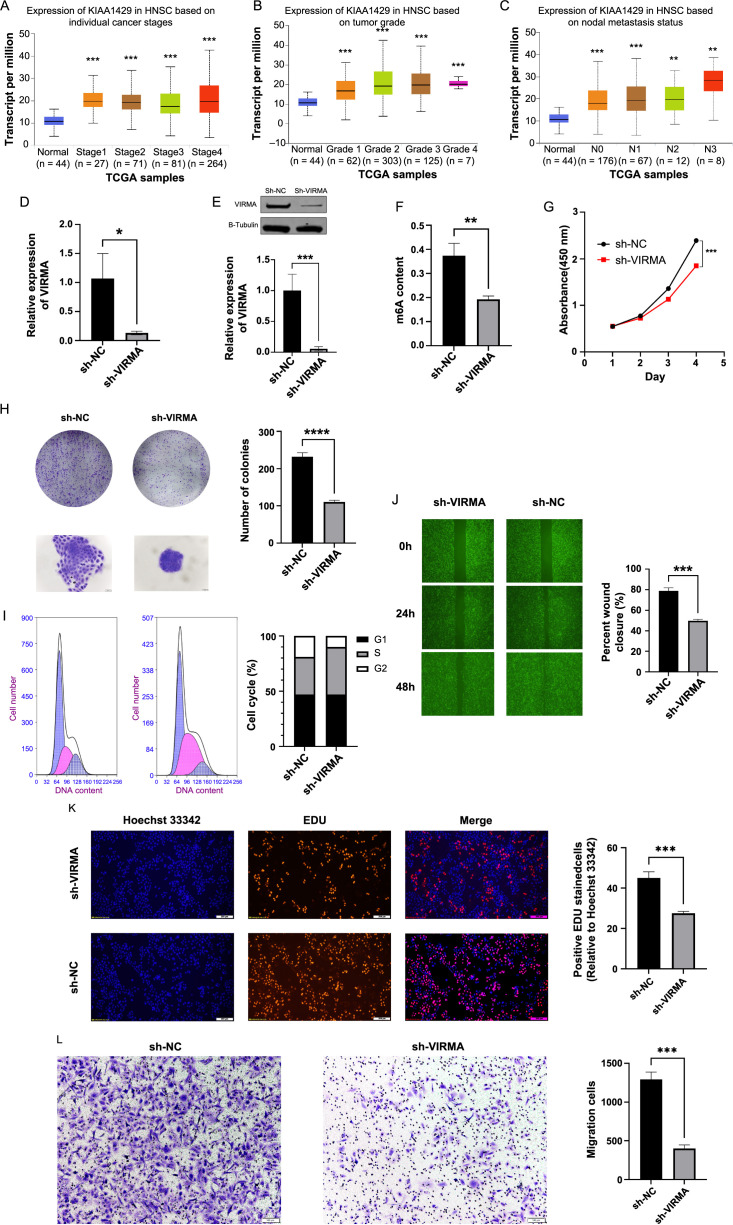
**Knocking down VIRMA may decrease the proliferation, invasion, and migration of HNSCC.** ****P < 0.0001, ***P < 0.001, **P < 0.01. (A) VIRMA expression was positively correlated with the cancer stage; (B) The levels of VIRMA were associated with tumor grade; (C) VIRMA expression in HNSCC and normal tissues according to nodal metastatic status; (D and E) Examination of the VIRMA expression level in SUNE-2 cells transfected with sh-VIRMA by RT-qPCR and western blot; (F) The concentration of m6A was decreased followed by the knockdown of VIRMA; (G) The proliferative capacity of SUNE-2 cells transfected with sh-VIRMA was assessed by measuring the absorbance at 450 nm via CCK8 assay; (H) The proliferative ability of the cells was determined by clonal formation assay, scale bar 50 um; (I) SUNE-2 cells were stained and cell cycle analysis by flow cytometry showed that SUNE-2 cells were mostly arrested in the S phase; (J) EdU-positive rate of SUNE-2 cells transfected with sh-VIRMA was detected by EdU assay, scale bar 200 µm; (K) Wound-healing assays were used to evaluate the migratory capacity of SUNE-2 cells after transfection with the indicated shRNA, scale bar, 500 µm; (L) Investigation of the effect of VIRMA decrement on the ability of SUNE-2 cells to invade via transwell invasion experiment, scale bar, 100 um. HNSCC: Head and neck squamous cell carcinoma; VIRMA: Vir-like m6A methyltransferase associated; RT-qPCR: Reverse transcription-quantitative polymerase chain reaction; CCK8: Cell counting kit-8.

To gain a deeper understanding of the importance of VIRMA in HNSCC, we analyzed VIRMA expression and cancer stage, tumor grade, and nodal metastasis by using the UALCAN online database. The amplification of VIRMA expression is evident during the progression of HNSCC from stage I to stage IV (Figure 3A). Analogously, the augmentation of VIRMA expression escalates with the advancement of HNSCC from grade 1 to grade 4, and increased nodal metastasis (Figure 3B and 3C). To corroborate these findings, we selected the SUNE-2 cell, which exhibited the highest VIRMA expression, for additional experimentation. VIRMA was knocked out using lentivirus, and the efficiency of this knockout was confirmed via RT-qPCR and western blot analyses (Figure 3D and 3E). To our delight, upon the knockdown of VIRMA, the levels of m6A experienced a decline, aligning perfectly with our anticipations (Figure 3F). To further investigate the role of VIRMA in HNSCC cell proliferation and metastasis, we assessed the proliferation capacity of HNSCC cells after VIRMA knockout using CCK-8, colony formation, and EDU assays, and measured the HNSCC cell cycle using flow cytometry. The results showed that after VIRMA knockout, cell proliferation, and colony formation were reduced (Figure 3G, 3H, and 3J), and HNSCC cells mostly stagnated in the S phase, which meant that the number of tumor cells dividing after VIRMA knockout was reduced (Figure 3I). Correspondingly, the migration and invasion prowess of HNSCC witnessed a reduction following the knockdown of VIRMA, as demonstrated by scratch and transwell assay (Figure 3K and 3L). These experimental results suggest that the reduction in VIRMA expression can inhibit the proliferation and metastasis of HNSCC.

**Figure 4.**
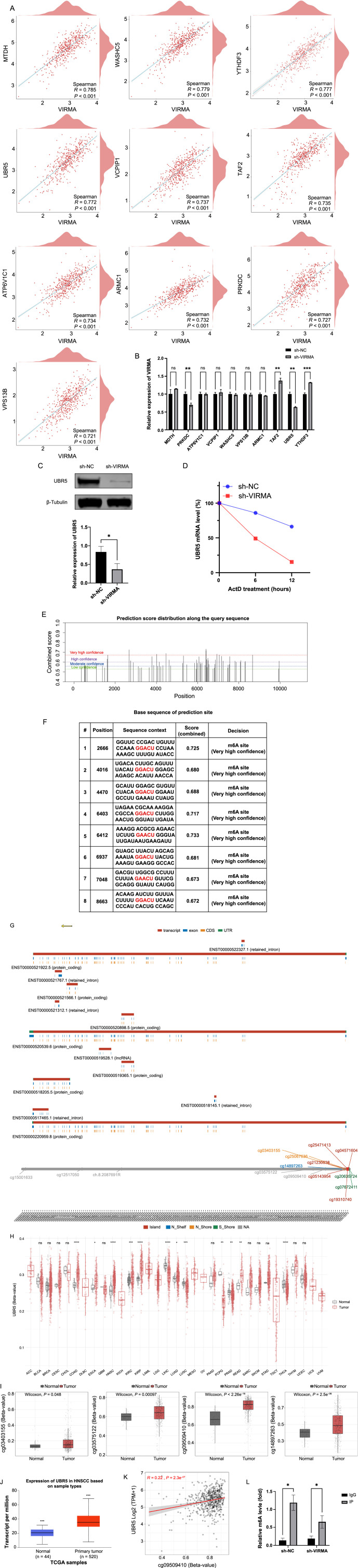
*** UBR5* regulation by VIRMA impacts tumor incidence and progression.** *P* > 0.05, *P < 0.05, **P < 0.01, ***P < 0.001, ****P < 0.0001. (A) A Spearman correlation analysis was performed between VIRMA and other genes in HNSCC to identify the top ten genes with the highest correlation coefficients; (B) The association between VIRMA and nine other genes was substantiated through RT-qPCR; (C) The protein expression of *UBR5* in sh-NC and sh-VIRMA groups was detected by western blot; (D) Degradation rate of *UBR5* mRNA in SUNE-2 cells treated with VIRMA and actinomycin D (10 µg/mL) at corresponding time points; (E) Potential loci for m6A modification within the genomic sequence of the *UBR5* gene; (F) Very high confidence nucleotide sequences for m6A modification sites within *UBR5*; (G) Genomic information of *UBR5*; (H) Expression of CPG-aggregated methylation in 33 cancers; (I) Differential CPG correlates of *UBR5* in normal tissue and HNSCC; (J) VIRMA expression was higher in tumor tissues than in normal tissues; (K) A positive correlation was found between CPG and the expression of *UBR5* in HNSCC; (L) Following VIRMA knockdown, the m6A modification level of *UBR5* mRNA decreased. HNSCC: Head and neck squamous cell carcinoma; VIRMA: Vir-like m6A methyltransferase associated; RT-qPCR: Reverse transcription-quantitative polymerase chain reaction; CpG: Cytosine-phosphate-Guanine; UBR5: Ubiquitin protein ligase E3 component N-recognin 5; CpG: Cytosine-phosphate-Guanine; sh-NC: The control group; sh-VIRMA: VIRMA knockdown group.

### *UBR5* regulation by VIRMA impacts tumor incidence and progression

To identify the downstream genes that VIRMA regulates to govern tumor occurrence and progression, we conducted Spearman correlation analysis on the RNA-seq data in the TCGA database and selected the top ten genes with the highest correlation coefficient. They are *MTDH, WASHC5, YTHDF3, UBR5, VCPIP1, TAF2, ATP6V1C1, ARMC1, PRKDC,* and *VPS13B* (Figure 4A). To verify this hypothesis, we conducted RT-qPCR and western blot verification, and the results showed that *UBR5* was most significantly reduced in the sh-VIRMA group (Figure 4B and 4C). The stability of *UBR5* mRNA was examined to clarify whether VIRMA enhances its expression by delaying RNA degradation. Indeed, *UBR5* mRNA showed accelerated degradation after VIRMA knockdown (Figure 4D). Subsequently, we analyzed the m6A modification sites and the base sequences of the modification sites within the *UBR5* genome (Figure 4E and 4F). Figure 4G presents the detailed genomic information of *UBR5* and methylation-associated CpGs. By integrating probe information with clinical data, we further explored the role of *UBR5* methylation in tumors. Compared to normal tissues, *UBR5* showed a substantial degree of hypermethylation in various tumor tissues, especially in HNSCC (Figure 4H and 4I). Furthermore, there was a positive correlation between the methylation level and the expression level of *UBR5* (Figure 4J and 4K). The m6A level of *UBR5* was significantly reduced after VIRMA knockdown, as confirmed by meRIP-qpcr verification (Figure 4L).

## Discussion

The study’s novel discovery is that VIRMA regulates the expression of *UBR5* via m6A-mediated mechanism, ultimately leading to the progression of HNSCC, suggesting that VIRMA has shown potential as a biomarker for the prognosis of HNSCC.

The entire process of messenger RNA methylation in human cells requiring VIRMA was first reported in 2015 [6]. As a virus homolog in fruit flies, virilizer plays a crucial role in sex-lethal splicing, the viability of males and females, and the capacity to generate eggs in embryonic development [15]. Within the nucleolus of human cells reside VIRMA and WTAP, sharing this location [16, 17]. VIRMA, the most substantial known constituent of the methyltransferase complex at 202 kDa, is composed of an N-terminal segment, denoted as N-VIRMA, and a C-terminal portion, commonly referred to as C-VIRMA. It starts in the SUN domain [18–20]. Reported that after the VIRMA knockdown, the m6A peak score decreased four times, which is more apparent than detected in human cells after the knockdown of METTL3 and METTL14 [7]. The constituents of m6A consist of “writers,” “erasers,” and “readers,” all of which have significant implications in cancer. Among them, VIRMA was found to occupy the largest known component in the readers, indicating that VIRMA might have various functions and play a crucial role in cancer pathways.

Li’s study [21] showed the differences in VIRMA expression between different tissues. The investigation uncovered that VIRMA is highly expressed in several malignancies, such as hepatocellular carcinoma (HCC), kidney chromophobe, HNSCC, lung adenocarcinoma, lung squamous cell carcinoma, colorectal cancer, rectal adenocarcinoma, breast invasive carcinoma, kidney clear cell carcinoma, and cholangiocarcinoma. VIRMA exhibits diminished expression in kidney renal papillary cell carcinoma, thyroid carcinoma, prostate adenocarcinoma, and uterine corpus endometrial carcinoma. However, the precise function of m6A methylation in the proliferation and metastasis of HNSCC cells is still unclear. In the current study, we confirmed that VIRMA is upregulated in HNSCC and further found that VIRMA is a potential prognostic factor affecting tumor growth and lymphatic metastasis in HNSCC. Subsequently, we examined the influence of VIRMA expression on the phenotypic characteristics of HNSCC cells. A reduction in VIRMA expression curtails the proliferation, migration, and invasion of HNSCC cells.

Being the most substantial known composing of the methyltransferase complex, VIRMA forms most of the m6A deposits on mRNA. We found a positive association between the expression of VIRMA and HNSCC and the level of m6A modification, which is consistent with the m6A methylation effect of VIRMA. The RNA methyltransferase VIRMA may regulate mRNA by m6A modification of target genes. However, the potential modification targets of VIRMA-regulated genes in HNSCC are still unclear. Therefore, we screened the top 10 with the highest correlation with VIRMA by analyzing the RNA-seq data in the TCGA database. Then, we verified this result using RT-qPCR and western blot. Moreover, we identified the m6A modification sites of *UBR5* using the SRAMP tool and smart app database, subsequently analyzing the methylation levels in both normal and tumorous tissues. Finally, it was proved that VIRMA regulates the expression of *UBR5* in an m6A-dependent manner, thus affecting the development of HNSCC.

Previous research has demonstrated that *UBR5* functions as an oncogene in the formation of tumors. For instance, the expression of *UBR5* in samples from patients with HCC significantly exceeded than observed in adjacent normal tissues. Increased *UBR5* expression levels were associated with advanced age among patients, higher tumor grading, the presence of lymph node metastasis, and a reduced overall survival duration [22]. The expression of *UBR5* is significantly elevated in both glioblastoma tissues and cells, and *UBR5* promotes the epithelial–mesenchymal transition of glioblastoma cells, thereby accelerating the invasion, proliferation, and migration of glioblastoma cells. Wu et al. [23] demonstrated that *UBR5* enhances glioblastoma cell migration and invasion by regulating the ECRG4/NF-κB signaling pathway. In addition, *UBR5* was found in nasopharyngeal carcinoma tissues. Compared to nearby normal tissues, the levels of UBR5 protein and mRNA were found to be increased, and *UBR5* also promoted the progression of nasopharyngeal carcinoma as a downstream gene of miR-135a-3p [24].

Our research is still in its early stages and has some limitations. First, *UBR5* was identified solely through database screening without the inclusion of high-throughput sequencing. Second, although we have demonstrated the impact of VIRMA on HNSCC by regulating the levels of *UBR5* and m6A, the potential role of reading proteins between VIRMA and *UBR5* remains unknown. Finally, our focus is limited to studying the VIRMA-mediated m6A modification of *UBR5*. We did not investigate the effect of *UBR5* on HNSCC phenotypes. These findings will guide our future endeavors as we plan to expand our research to explore relationships between other reading proteins and target genes while delving into the underlying mechanisms connecting m6A modifying enzymes, reading proteins, and target genes.

## Conclusion

Our study demonstrated that VIRMA is crucial in increasing m6A levels and promoting *UBR5* expression in HNSCC. Knockdown of VIRMA leads to decreased m6A levels and *UBR5* expression, resulting in inhibited proliferation and migration of HNSCC. This study revealed a novel mechanism of VIRMA in HNSCC, providing potential targets and new directions for treating this disease.
